# Transposon Domestication versus Mutualism in Ciliate Genome Rearrangements

**DOI:** 10.1371/journal.pgen.1003659

**Published:** 2013-08-01

**Authors:** Alexander Vogt, Aaron David Goldman, Kazufumi Mochizuki, Laura F. Landweber

**Affiliations:** 1 Institute of Molecular Biotechnology of the Austrian Academy of Sciences (IMBA), Vienna, Austria; 2 Department of Ecology and Evolutionary Biology, Princeton University, Princeton, New Jersey, United States of America; Baylor College of Medicine, United States of America

## Abstract

Ciliated protists rearrange their genomes dramatically during nuclear development via chromosome fragmentation and DNA deletion to produce a trimmer and highly reorganized somatic genome. The deleted portion of the genome includes potentially active transposons or transposon-like sequences that reside in the germline. Three independent studies recently showed that transposase proteins of the DDE/DDD superfamily are indispensible for DNA processing in three distantly related ciliates. In the spirotrich *Oxytricha trifallax*, high copy-number germline-limited transposons mediate their own excision from the somatic genome but also contribute to programmed genome rearrangement through a remarkable transposon mutualism with the host. By contrast, the genomes of two oligohymenophorean ciliates, *Tetrahymena thermophila* and *Paramecium tetraurelia*, encode homologous PiggyBac-like transposases as single-copy genes in both their germline and somatic genomes. These domesticated transposases are essential for deletion of thousands of different internal sequences in these species. This review contrasts the events underlying somatic genome reduction in three different ciliates and considers their evolutionary origins and the relationships among their distinct mechanisms for genome remodeling.

## Introduction

A transposon rearranges its host's genome when it moves from one genomic locus to another. When they invade coding or regulatory regions, transposons can alter gene expression. Furthermore, transposon-induced DNA double-strand breaks can cause chromosomal rearrangements and subsequent aneuploidy. Thus, transposons were long considered as harmful and selfish “junk DNA” [1]. However, because most eukaryotic genomes have maintained transposons and transposon-derived DNA throughout the course of evolution, it is possible that they sometimes confer an adaptive benefit to the host [2]. Because maintenance in the host genome also benefits the transposon, this would be a form of mutualism. Transposons can also accelerate genome evolution by fabricating new sequences and facilitating genome rearrangement.

Often the host manages to recruit or “domesticate” transposon-encoded genes and repurpose them for new host functions [3], [4]. A domestication event typically alters the transposon-derived sequence, curtailing its mobility. Thus it no longer meets the functional definition of a transposon. A famous example in jawed vertebrates is the evolution of the *RAG1* gene from a *Transib*-like element. Now a key component in V(D)J recombination, it is responsible for cutting and rejoining *V*, *D*, and *J* segments [5],[6]. As this process is indispensable for maturation of B and T cells, the *RAG1* gene domestication enabled the evolution of adaptive immunity [6]. Other examples of domesticated transposases include the yeast *Klyveromyces lactis* α3 MULE transposase-like protein, which enables mating-type switching [7]. In addition, *C. elegans* HIM-17 is a domesticated P-element–like transposase that is essential for double-strand break and chiasma formation during meiosis, as well as for the accumulation of histone H3 methylation at lysine 9 on meiotic prophase chromosomes [8]. Therefore, transposon domestication is widespread, and transposons supply toolkits for host cells to evolve new functions. However, the processes by which transposons become domesticated can vary.

Recently, three groups discovered crucial roles for transposase-related proteins in large-scale genomic rearrangements in three different ciliate species [9]–[11]. Paramecium and Tetrahymena (both Oligohymenophorea) use single-copy domesticated transposase genes for genomic rearrangements. Curiously, *Oxytricha trifallax*, a member of a different, deeply diverged ciliate class (Spirotrichea), requires instead the expression of thousands of active transposase genes that still reside in intact—and potentially active—transposons. Therefore, comparison of these different transposon-derived systems offers a unique opportunity to put in a broad evolutionary context two different scenarios for the recruitment, or “exaptation” [12], of either active or modified transposon functions in the emergence of new biological pathways.

## Programmed Genome Remodeling in Ciliates

**Figure 1:**
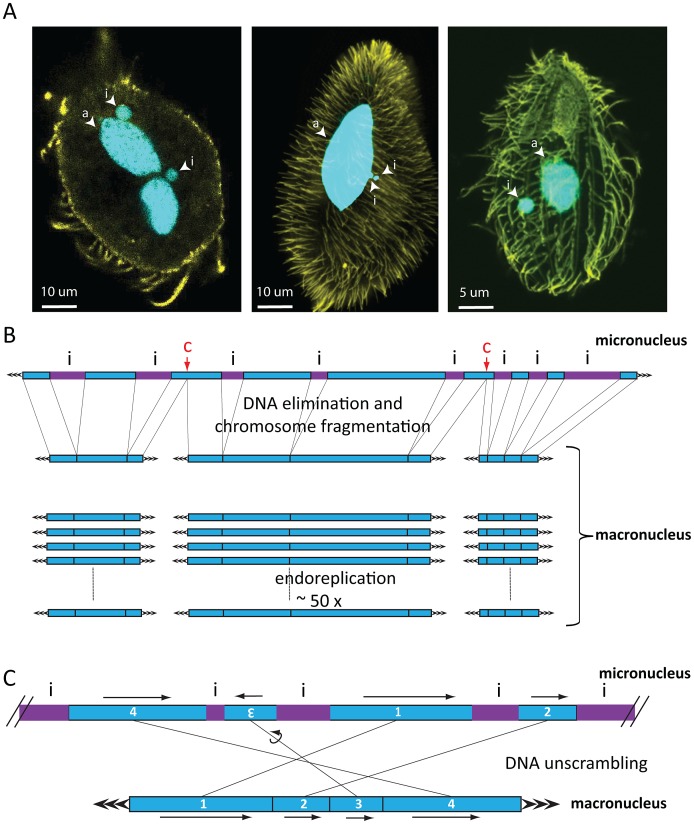
Nuclear dimorphism and genome rearrangements in ciliates. A) From left to right: *Oxytricha trifallax*, *Paramecium tetraurelia*, *Tetrahymena thermophila*. DNA is shown in cyan, yellow represents tubulin staining. Images were kindly provided by Wenwen Fang (Princeton University, Princeton), Kensuke Kataoka (IMBA, Vienna), and Janine Beisson (CNRS, Gif sur Yvette). Abbreviations: i = micronucleus, a = macronucleus. In *Oxytricha trifallax*, two lobes of a macronucleus are connected by a thin nuclear bridge (not visible in the image). B) Genome rearrangements in all ciliates shown include elimination of micronucleus (MIC)-limited sequences (i, purple IES) and chromosome breakage, which in Tetrahymena occurs at specific chromosome breakage sites (labeled c). After religation of the flanking macronuclear (MAC) sequences, Tetrahymena chromosomes undergo endoreplication to produce 50 identical copies. C) DNA unscrambling in Oxytricha involves the reshuffling and occasional inversion of precursor micronuclear (MIC) sequences (numbered blue boxes) to assemble them in the correct macronuclear order.

Ciliates are microbial eukaryotes and members of the Alveolata that include dinoflagellates and apicomplexan parasites [13]. A common feature is nuclear dimorphism, with two types of nuclei in the same cytoplasm. The larger DNA-rich somatic macronucleus [14] provides most gene expression during vegetative growth. The smaller germline micronucleus [14] is diploid and transcriptionally active mainly during conjugation. Actual numbers of macronuclei and micronuclei vary among ciliate species [14]. *Oxytricha trifallax* and *Paramecium tetraurelia* each have one macronucleus and two micronuclei in interphase vegetative cells, whereas *Tetrahymena thermophila* has one macronucleus and one micronucleus (Figure 1A) [14]. During asexual division, both nuclei divide; whereas during sexual conjugation, the zygotic micronucleus gives rise to both a new macronucleus and a new micronucleus, supplying the next generation with all its genetic information. However, the macronucleus and micronucleus differ substantially in their genetic content because the somatic genome undergoes an elaborate cascade of events that produces a new macronucleus from the zygotic micronucleus, after cell mating [15], [16].

Genome rearrangements during macronuclear development in ciliates delete large portions of germline DNA and consequently produce greater numbers of small, somatic chromosomes (on the order of 16,000 different types in *Oxytricha trifallax* [17]) than their longer, germline chromosomes. There is considerable variation in this process between major lineages. Macronuclear chromosomes in oligohymenophorean ciliates have an average size of 300 kbp in Tetrahymena and 800 kbp in Paramecium [18]–[20]. In contrast, spirotrichous ciliates like Oxytricha typically have gene-sized nanochromosomes in the macronucleus, which average just 3.2 kbp including short telomeres, and 90% encode just a single gene [17]. DNA elimination discards between 20% (Tetrahymena) and ∼95% (Oxytricha) of the entire germline genome during macronuclear development [16]. In some spirotrichs, as well as phyllopharyngeans [21], rearrangements in some loci also require DNA unscrambling (Figure 1C). These often complex events reorder gene pieces in the micronucleus by translocation or inversion to assemble coding information in the macronucleus [22].

Despite the genome downsizing via DNA elimination, the macronucleus contains a greater quantity of DNA than the micronucleus. This is because macronuclear chromosomes undergo endoreplication to amplification levels that typically range from 50-fold in Tetrahymena to 800-fold in Paramecium and up to 2,000-fold in Oxytricha [16]. This review focuses on DNA elimination. For a review of genome unscrambling and the role of RNA-regulated epigenetic effects in this process, as well as DNA amplification, we refer the reader to Nowacki et al. (2011) [22], and for a summary of the relationship among ciliate species with available genome information and amplification levels, we refer the reader to Figure 2 of Swart et al. (2013) [17]. Although DNA elimination is common to most ciliates, recent studies that we describe below revealed a dependence on strikingly different groups of transposase-related proteins for DNA elimination in different classes of ciliates [9]–[11].

## Deletion of Germline-Limited Sequences in Oxytricha

**Figure 2:**
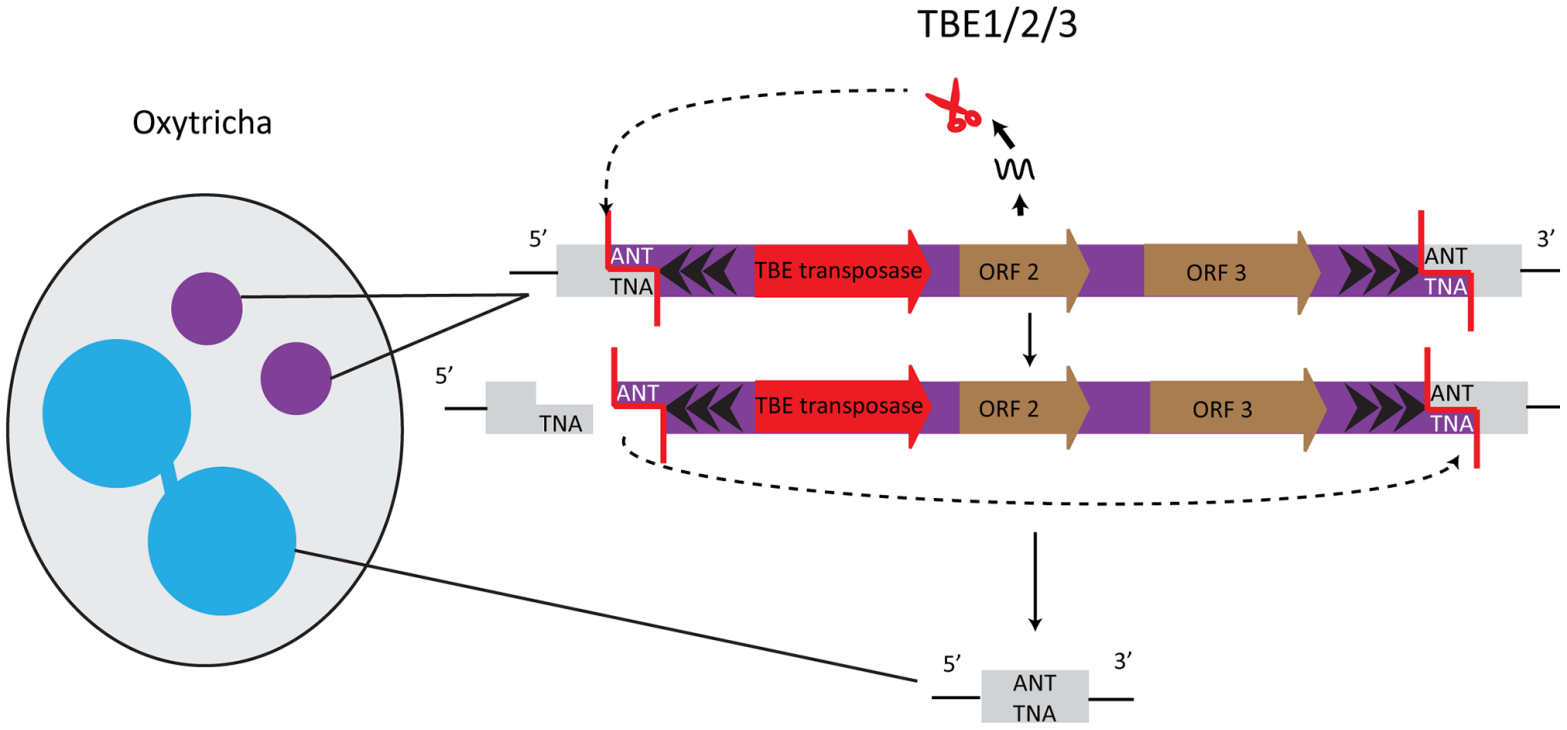
TBE transposases in Oxytricha are germline-limited sequences and they participate in their own removal. The encoded transposases of the Tc1/*mariner* family have a DDE catalytic motif. Cleavage of the germline-limited sequences starts with a 3 nucleotide 5′ overhang at an ANT recognition site; the second target site serves as the integration site [24].

Elimination of germline-restricted DNA sequences usually occurs at precise, nucleotide-level resolution in *Oxytricha trifallax*. One well-studied example of precisely removed germline-limited sequences are the Tc1*/mariner* transposons of the TBE (telomere-bearing element) class, which are present in thousands of copies in the micronucleus [23] and occupy roughly as much of the micronuclear genome as its estimated coding content. TBE terminal regions possess inverted repeats, with the most distal 17 bp composed of telomeric repeats ((G_4_T_4_)_2_G)*_n_*, and the elements are flanked by a 3 bp 5′-ANT-3′ target site duplication (Figure 2). TBE excision precisely removes one target site repeat, thereby restoring functional open reading frames (ORFs) even when TBEs interrupt protein-coding regions in the micronucleus. Mechanistically, it is likely that introduction of a double-stranded break (DSB), creating a 3 nt 5′ protruding end on one side of the transposon, initiates excision. The other target site serves as an “integration site” so that TBEs excise in a circular form, the TBE ring degrades, and macronuclear DNA religates [24] (Figure 2).

One of the TBE-encoded genes encodes a protein belonging to the DDE transposase superfamily, suggesting involvement of this enzyme in the transposon's own removal [24], [25] (Figure 2). Furthermore, all three TBE transposon ORFs appear to be under purifying selection, which initially hinted at an important function of the transposases [26], [27]. *Oxytricha trifallax* has three different types of TBE transposons: TBE1, TBE2, and TBE3. The transposases encoded by these elements share ≥83% similarity at the protein level, and all three types of transposases are specifically expressed during macronuclear development when DNA rearrangements occur. RNAi against all three groups of TBE transposases in unison (but not individually) results in severe defects in elimination of both TBE transposons and non-TBE micronucleus-limited elements, as well as an accumulation of high molecular weight DNA [10]. These results lead to two non–mutually exclusive hypotheses: first, that TBE transposases act redundantly in excising both the transposons that encode them and other micronucleus-limited sequences (“internal eliminated sequences” or IESs); and second, because this experiment silenced thousands of paralogs that occupy a significant fraction of the germline genome, it suggests that a massive quantity of transposase may be required for Oxytricha genome rearrangement [10].

## DNA Deletion in Paramecium and Tetrahymena

**Figure 3:**
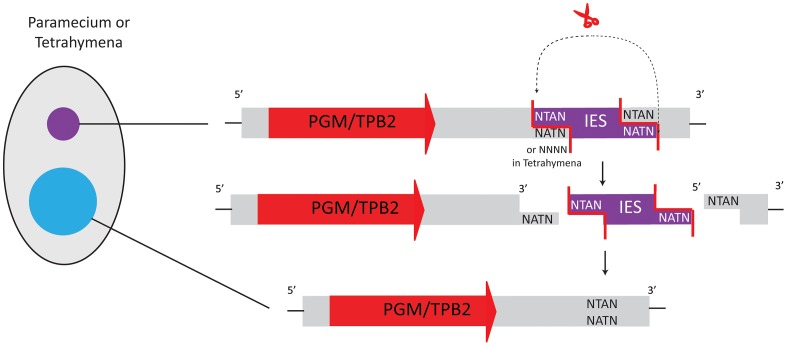
Transposases in Tetrahymena and Paramecium belong to the PiggyBac family. As domesticated transposases, they are present as single-copy genes in the micronuclear and macronuclear genomes. After expression from the somatic genome, they facilitate IES excision from the new macronuclear genome. IES removal occurs via a 4-base 5′ protruding end. In Paramecium, all deleted sequences have a TA dinucleotide at both boundaries, whereas Tetrahymena displays no consensus sequence.

*Paramecium tetraurelia* has two types of eliminated sequences. Most repetitive micronucleus-limited sequences, similar to minisatellites or transposons, are eliminated imprecisely [16], [28]. In contrast, removal of approximately 45,000 different non-repetitive, single-copy IESs occurs precisely [29]. Though both types of eliminated sequences are removed reproducibly, elimination can produce microheterogeneity within a few base pairs [29]. Paramecium IESs are flanked by a 5′-TA-3′ dinucleotide, part of a weakly conserved 8 bp sequence with similarity to the recognition sequence of some Tc1*/mariner* transposases [30], [31]. This led Klobutcher and Herrick in an elegant model [25] to propose that some Paramecium IESs are remnants of Tc1*/mariner* transposons. However, IES excision starts with a double-strand break that produces a 4-base 5′-overhang [32] (Figure 3), whereas Tc1*/mariner* transposases yield 2-base 3′-overhangs [33], so this model would require either extensive modification of the original transposase mechanism during domestication, or recruitment of different enzymes.

IES elimination in *Tetrahymena thermophila* [34] also produces double-strand breaks with 4-base 5′-overhangs. Tetrahymena removes ∼6,000–9,000 different IESs from its developing macronucleus [35], [36], an order of magnitude fewer IESs than Paramecium. Tetrahymena IESs are typically larger than in Paramecium (from ∼200 bp to >20 kbp). Most are eliminated imprecisely, leaving heterogeneity in the resulting macronuclear sequences. Hence they rarely interrupt exons, with few exceptions [37] that would be weakly conserved regions. Some IESs do bear similarity to Tc1/*mariner* transposons or non-LTR retrotranposons [19], [38]. Although no obvious consensus sequence exists at Tetrahymena IES boundaries, the DNA double-strand breaks (DSB) in both Paramecium and Tetrahymena produce 4-base 5′-overhangs [32], [34]. This suggested similar enzymes for DNA elimination and led researchers to search for PiggyBac transposases that could produce such ends.

Indeed, the macronuclear genomes of both species contain genes derived from PiggyBac family transposases, and Baudry et al. and Cheng et al. independently showed that a transposase of the PiggyBac family plays a crucial role in DNA elimination during maturation of the macronuclear genomes in Paramecium and Tetrahymena [9], [11]. These ciliate transposon-derived proteins are called Pgm (PiggyMac) in Paramecium and Tpb2p (Tetrahymena PiggyBac-like transposase 2) in Tetrahymena. In both Paramecium and Tetrahymena, silencing of the respective PiggyBac transposase-like genes by RNAi inhibits the processes of DNA elimination and macronuclear development [9], [11]. Both Pgm and Tpb2p have a predicted catalytic domain with conserved DDD residues, similar to PiggyBac transposases. *In vitro* studies with Tpb2p recombinantly expressed in *E. coli* revealed that Tpb2p produces a double-strand break leaving a 4-base 5′ protruding end, which correlates with the typical cleavage signature of canonical PiggyBac transposases [11], [39] and the observed form of DSB during DNA elimination *in vivo* [34] (Figure 3). Therefore, Pgm and Tpb2p are probably the enzymes responsible for catalyzing DNA excision during DNA elimination in Paramecium and Tetrahymena, respectively.

**Figure 4:**
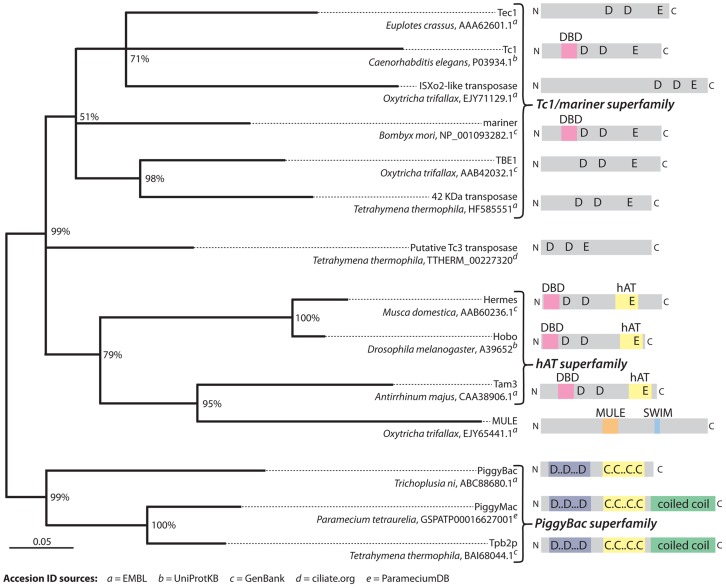
Phylogenetic analysis of representative transposases of the DDE/DDD superfamily. This tree supports the conclusion that TBE elements belong to the Tc1/*mariner* superfamily of transposons and also that there appear to be TBE-like elements present in Tetrahymena (labeled “42 kDa transposase”). Additionally, this analysis supports the conclusion that the two PiggyBac-like transposases, Pgm and Tpb2p, in Paramecium and Tetrahymena are homologous to each other. The grouping of the MULE family representative [17] within hAT transposases is unexpected [47] and possibly the result of an alignment artifact due to its disproportionately long sequence. Recently discovered Paramecium transposases Sardine, Thon, AnchoisA, and AnchoisB [29] were omitted because their inclusion in the analysis significantly lowered confidence scores for a majority of branches. The tree was created with MRBayes phylogenetic inference software [48] using the alignment shown in Dataset S1, which was edited to remove regions with gaps in the consensus sequence. The phylogeny was generated using a mixed amino acid substitution model and invariable gamma distribution rate model over 200,000 iterations with a burn-in of 25%. Branch confidence values represent conditional probabilities generated by the Bayesian inference process. The scale bar corresponds to 0.05 expected substitutions per site of the unmasked alignment positions. Domain and motif annotations were produced using the Pfam web server [49].

Both Pgm and Tpb2p localize to the newly developing macronucleus during DNA elimination. Tpb2p localizes to the subnuclear heterochromatin bodies where DNA elimination is thought to occur [9], [11]. These heterochromatin structures contain heterochromatin-specific histone modifications, tri-methylated histone H3 lysine 9 (H3K9me3) and lysine 27 (H3K27me3), and the chromodomain protein Pdd1p [40]–[42]. Localization of Tpb2p to these structures could be mediated by an interaction with some of these or other heterochromatin components. Pgm and Tpb2p share a predicted zinc finger domain and coiled-coil domain (Figure 4) that may directly interact with some of the heterochromatin components. Because heterochromatin is specifically established on Tetrahymena IESs prior to their elimination via an RNAi-related pathway [41], [42], the heterochromatin interaction of Tpb2p, and possibly other PiggyBac transposase-like proteins, may restrict their action to programmed deleted sequences.

In addition to the association of PiggyBac transposase-like proteins with heterochromatin, their enzymatic preference for certain DNA sequences may both facilitate IES elimination and constrain it evolutionarily. For instance, the 5′-TA-3′ dinucleotide that flanks Paramecium IESs is also the smallest sequence recognized by the canonical PiggyBac transposase, whose optimal recognition sequence is 5′-TTAA-3′ [39]. Moreover, recombinant Tpb2p in solution can specifically cleave a dsDNA oligonucleotide containing 5′-TTAA-3′ before the first T [11] and cleaves the left boundary of a deleted region in Tetrahymena (5′-AGTGAT-3′) between the first A and G, when this motif is placed in the middle of an otherwise randomly designed 50 bp dsDNA oligonucleotide [11]. Therefore, although a 5′-TA-3′ is not necessary for Tpb2p cleavage, the enzyme probably recognizes limited sequence context. However, it is unlikely that primary DNA sequence is the sole determinant. Most likely, both heterochromatin interactions of Tpb2p and Pgm and their preference for certain DNA sequences determine cleavage sites for DNA elimination.

## Transposon Domestication versus Mutualism: Possible Evolutionary Origins

Although Pgm and Tpb2p are similar to PiggyBac transposases, they are not present in active transposons and their genes are single-copy in the macronucleus. Thus, they are classic examples of transposon domestication by the host genome to mediate a new function—in this case, DNA elimination, consistent with [25]. Pgm and Tpb2p share 30% global identity, which either suggests a single domestication of a PiggyBac-like transposase in their oligohymenophorean ancestor or independent recruitments of related transposases. No PiggyBac-like transposase has been found in Oxytricha. After the Paramecium and Tetrahymena lineages separated, the domesticated transposases accumulated substitutions that could contribute to the differences in their DNA deletion pathways, as well as the apparent promiscuity of Tpb2p at Tetrahymena IES boundaries.

Recruitment of a single domesticated transposase in Oligohymenophorea is in sharp contrast to Oxytricha's distributed system that appears to enlist an army of thousands of TBE transposases that still reside in potentially active Tc1/*mariner* transposons (Figure 4) and occupy a significant fraction of the germline genome (the transposon “bloom” phase of Klobutcher and Herrick's model for IES origins from transposons [25]). Oxytricha macronuclear development requires hundreds of thousands of rearrangement events, which may explain its need for increased transposase participation. The greater complexity of genome rearrangements does not, however, explain why Oxytricha should recruit undomesticated transposases to facilitate genome rearrangement. This strategy may be easier to evolve, as active transposons would multiply in number, up to the ceiling tolerated by its host, which would ensure production of an ample quantity of transposase, in part because these enzymes also facilitate elimination of their parent transposons. This achieves a mutualistic evolutionary balance between the host and its resident germline transposons [43]. It also wonderfully displays a functional and essential role for this otherwise dispensable portion of the micronuclear genome [10]. During the divergence of spirotrichs, TBE transposases may have gained promiscuity and acquired the ability to excise off-target DNA sequences [24], [25]. Additionally, only DNA insertions with the ability to be excised by TBE transposases or other active enzymes would have been tolerated in the germline over time, and thus could accumulate. Such a mutualism [43] would have allowed not only the accumulation of germline transposons but also the production of a sufficient quantity of transposase protein to facilitate Oxytricha's elaborate process of genome remodeling and also exclude these active transposons from the soma. These requirements could have provided the selective pressure to maintain high transposon copy numbers to facilitate DNA elimination. Conversely, the DNA elimination events that domesticated PiggyBac transposases facilitate in Paramecium and Tetrahymena do not require maintenance of germline transposons. This striking difference in two evolutionary lineages separated by over a billion years may have been exaggerated over time by an evolving trend in the Oxytricha lineage to eliminate and rearrange considerably more of its micronuclear genome.

The ostensible similarities and likely homology between PiggyBac and TBE transposons (Figure 4) belie their differences. How did different ciliate lineages acquire different types of transposases and coevolve such different strategies between the transposons and their hosts to mediate different pathways of genome differentiation? Because oligohymenophorean and spirotrich ciliates are evolutionarily more distant from each other than plants and animals, a plausible explanation for the recruitment of different types of transposases for DNA elimination pathways in these distant ciliates is independent origins. However, it is also possible that the mutualistic system in Oxytricha may have predated DNA elimination by a domesticated transposase. A later transposon-domestication event or events that resulted in a high quantity of active transposase in the ancestral oligohymenophorean lineage could have lessened the dependency on feral transposons distributed throughout the genome. The modern piggyBac-like element in Paramecium and Tetrahymena might be a relic from a transposon that was initially maintained in the micronucleus by a mutualistic system more like Oxytricha's, and then later a copy of its transposase gene could have accidently lost the signals for DNA deletion and become a resident of the macronucleus as well, where it accumulated additional substitutions. Then this PiggyBac transposase, if expressed at sufficiently high levels, could have taken over the former roles of TBE or other transposases, reducing the levels of purifying selection that acted on the germline transposases until they became redundant with the function of the domesticated transposase. This relaxation of constraints on germline transposons would have permitted them to adapt or ameliorate to the background of micronuclear-limited DNA, scattering transposon remnants in the micronuclear genome, until most were eventually unrecognizable [25]. Accordingly, sequences related to TBE transposases are present in the Paramecium [29] and Tetrahymena micronuclear genomes, and some have functional open reading frames that maintain the DDE catalytic triad (Anchois, Thon, and Sardine in Paramecium [29] and the Tetrahymena sequence labeled “42 kDa transposase” in Figure 4). Therefore, these DNA sequences could be remnants from a TBE mutualistic system, and the minimal conservation suggests the possibility that TBE transposases could still contribute some role to DNA elimination in oligohymenophoreans. In this context, it would be fruitful to study the function of these newly discovered TBE transposase genes, as well as other newly discovered transposase-related genes in the Oxytricha macronucleus [17].

## Conclusions

The roles of transposase proteins in programmed DNA rearrangements are just coming to light. Both structural and more functional studies are needed to understand how TBE and PiggyBac transposases interact with chromatin and induce DNA double-strand breaks. DNA elimination events, initiated by double-strand breaks, must be swiftly followed by DSB repair. Knowledge that the DNA elimination pathways in Paramecium and Tetrahymena require nonhomologous end joining (NHEJ) DSB repair machinery [44], [45] raises questions about how transposases interact with the NHEJ machinery and how they cooperatively regulate DNA elimination. In Oxytricha and other species with scrambled genes, another key set of questions is how the RNA templates [22] that provide the reordering information guide the transposases and other rearrangement machinery to form the proper religated junctions. From an evolutionary point of view, broader phylogenetic surveys are necessary to understand how two such distant groups of ciliates evolved such different DNA deletion systems, dependent on PiggyBac and TBE transposases, respectively. Because these two lineages represent just a modest fraction of ciliate biological diversity, and because some level of DNA elimination may be ancestral to ciliates [46], it would be tremendously valuable to investigate the functional and evolutionary relationships among transposases and DNA elimination events in different, deeply divergent groups of ciliates. Such comparative and functional studies are needed to achieve a better natural history of transposase recruitment and the forces of mutualism versus domestication on an evolutionary timescale.

## Supporting Information

Dataset S1 An amino acid sequence alignment of representative transposases of the DDE/DDD superfamily used to generate the phylogeny presented in Figure 4. The alignment was produced using the MUltiple Sequence Comparison by Log-Expectation (MUSCLE) web server (http://www.ebi.ac.uk/Tools/msa/muscle/). The masking sequence indicates alignment positions used for phylogenetic analysis. (FAS)
